# Development of a Clinical Decision Support Tool to Implement Asthma Management Guidelines in Pediatric Primary Care: Qualitative Study

**DOI:** 10.2196/65794

**Published:** 2025-03-18

**Authors:** David A Fedele, Jessica M Ray, Jaya L Mallela, Jiang Bian, Aokun Chen, Xiao Qin, Ramzi G Salloum, Maria Kelly, Matthew J Gurka, Jessica Hollenbach

**Affiliations:** 1 Center for Healthcare Delivery Science Nemours Children's Health Jacksonville, FL United States; 2 Department of Health Outcomes and Biomedical Informatics University of Florida Gainesville, FL United States; 3 Department of Clinical & Health Psychology University of Florida Gainesville, FL United States; 4 Department of Pediatrics University of Florida Gainesville, FL United States; 5 Department of Public Health Sciences University of Virginia Charlottesville, VA United States; 6 Asthma Center Connecticut Children's Medical Center Hartford, CT United States; 7 Department of Pediatrics UConn Health Farmington United States

**Keywords:** clinical decision support, asthma, primary care, guidelines, pediatric, asthma care, morbidity, health information technology, electronic health record, EHR, user-centered design, inductive approach, digital health, health technology

## Abstract

**Background:**

There is a longstanding gap between national asthma guidelines and their implementation in primary care. Primary care providers (PCPs) endorse numerous provider and practice or clinic-related barriers to providing guidelines-based asthma care. To reduce asthma morbidity in primary care, PCPs need access to tools that facilitate adherence to national guidelines, which can be delivered at the point of care, are minimally burdensome, and fit within the clinic workflow. Clinical decision support (CDS) tools are health IT systems that can be housed in the electronic health record (EHR) system.

**Objective:**

This study aimed to follow user-centered design principles and describe the formative qualitative work with target stakeholders (ie, PCPs and IT professionals) to inform our design of an EHR-embedded CDS tool that adheres to recent, significant changes in asthma management guidelines.

**Methods:**

Purposive sampling was used to recruit three separate subgroups of professionals (n=15) between (1) PCPs with previous experience using a paper-based CDS tool for asthma management, (2) PCPs without previous experience using CDS tools for asthma management, and (3) health care IT professionals. The PCP interview guide focused on their practice, familiarity with national asthma guidelines, and how a CDS tool embedded in the EHR might help them provide guideline-based care. The health care IT professional guide included questions on the design and implementation processes of CDS tools into the EHR. Qualitative data were audio-recorded, transcribed, and then analyzed using an inductive approach to develop themes.

**Results:**

Themes were organized into 2 domains, current practice and CDS tool development. The themes that emerged from PCPs included descriptions of assessments conducted to make an asthma diagnosis, previous attempts or opportunities to implement updated national asthma guidelines, and how a CDS tool could be implemented using the EHR and fit into the current asthma management workflow. The themes that emerged from health care IT professionals included processes used to design CDS tools and strategies to collect evidence that indicated a tool’s value to a practice and the broader health system.

**Conclusions:**

In this study, user-centered design principles were used to guide a qualitative study on perceived barriers and facilitators to a primary care–based, EHR-integrated asthma CDS tool. PCPs expressed their interest in adopting an asthma CDS tool that was low burden and efficient but could help them adhere to national asthma guidelines and improve clinic workflow. Similarly, health care IT professionals perceived an asthma CDS tool to be useful, if it adhered to EHR design standards. Implementation of a CDS tool to improve adherence of PCPs to recently updated national asthma guidelines could be beneficial in reducing pediatric asthma morbidity.

## Introduction

Asthma is the most common childhood chronic illness in the United States, affecting >5 million children [1], and is a leading cause of emergency department and urgent care visits [2], school absences [3,4], and reduced quality of life among youth [5,6]. The National Asthma Education and Prevention Program (NAEPP) guidelines are designed to prevent and reduce pediatric asthma morbidity via disseminating state of the science guidance to health care providers on asthma diagnosis and management [7]. NAEPP guidelines call for health care providers to regularly adhere to 4 pillars of asthma management—assessment of asthma control, provision of asthma education to families (eg, written asthma treatment plans), assessment of environmental triggers, and following pharmacological therapy recommendations [4]. Notably, NAEPP guidelines were recently updated in 2020 to include single maintenance and reliever therapy (SMART), a paradigm-shifting change to recommendations for the management of youth with persistent asthma [7].

Use of NAEPP guidelines improves asthma control, reduces the likelihood of exacerbations, and decreases health care usage [4,7-9]. However, there is a persistent gap between NAEPP guidelines and their implementation in practice, particularly among primary care providers (PCPs) [10,11]. PCPs are the frontline health care providers for youth with asthma and provide most ambulatory care [12,13]. Suboptimal adherence among PCPs to national asthma guidelines is documented in numerous studies [10,12,14,15]. For example, a chart review study of a large cohort of primary care practices found that only 9% of PCPs used a validated asthma control tool, just 6% provided an asthma treatment plan, and 30% failed to prescribe anti-inflammatory medication when indicated [16]. This pattern of findings is mirrored in recent results from the National Asthma Survey of Physicians [12].

The considerable gap between NAEPP guidelines and their implementation in primary care are driven by provider and practice or clinic-related factors [12]. PCPs encounter numerous barriers to providing guidelines-based asthma care, including time constraints, lack of familiarity and knowledge of the guidelines, and low self-efficacy surrounding guideline implementation [17,18]. Furthermore, primary care clinics are commonly characterized as suffering themselves from “information chaos” with limited administrative bandwidth for comprehensive, evidenced-based interventions [19]. To reduce asthma morbidity in primary care, PCPs need access to tools that facilitate adherence to national guidelines, which can be delivered at the point of care, are minimally burdensome, and fit within the clinic workflow [15,20,21].

Clinical decision support (CDS) tools are health IT systems that can be housed in the electronic health record (EHR) system and be effective in improving provider adherence to guidelines and patient outcomes [22,23]. CDS tools assist providers in patient-level clinical decision-making (eg, asthma diagnosis and severity designation) at the point of care via collecting relevant data, synthesizing information, and providing real-time recommendations, thereby reducing provider burden and complexity surrounding adherence and implementation of guidelines [24]. CDS tools were shown to be the most promising method for modifying provider behavior in a systematic review of 68 intervention studies on health care provider adherence to national asthma guidelines [14]. There is a critical need for the creation of a CDS tool for pediatric asthma that attends to the needs of PCPs, especially in the current era of recently updated NAEPP guidelines [21].

While paper-based CDS tools have been shown to increase PCP adherence to NAEPP guidelines and reduce asthma-related morbidity including the number of outpatient visits, hospitalizations, and emergency department visits [25-27], the ubiquity of EHRs in delivery and documentation of primary care, including for asthma management [28], provides the opportunity to provide real-time decision support within the electronic workflow. In addition, electronically presented CDS may support better adherence due to automatic provision of guidance and patient-specific suggestions to support decision-making [29]. While recent work provides key guidance to support implementation of the recent guidelines into electronic CDS, further work is necessary to understand local contexts and clinician behaviors that will support successful intervention development and implementation [30]. This study describes our formative qualitative work with target stakeholders (ie, PCPs and IT professionals) to inform our design of an EHR-embedded CDS tool that adheres to the 4 pillars of asthma management and is congruent with recent, significant changes in NAEPP guidelines [7].

## Methods

### Overview

We used purposive sampling to recruit three separate subgroups of professionals between January and March 2023, that are (1) PCPs with previous experience using a paper-based CDS tool for asthma management, (2) PCPs without previous experience using CDS tools for asthma management, and (3) health care IT professionals. Participants were recruited through study announcement emails to department listservs and brief presentations to raise awareness of the study during department meetings. In total, 15 individuals agreed to participate. We conducted 2 focus groups with PCPs (n=5 and n=4 participants, respectively), 1 health care IT professional focus group (n=2), 3 PCP individual interviews, and 1 health care IT professional individual interview. Some participants had a previous professional relationship with the study team (eg, had participated in previous studies). The first focus group was with PCPs (n=5) who had previous experience using Easy Breathing, a validated paper-based CDS tool for asthma management that translates NAEPP guidelines into a usable, efficient, and effective format [10,11,25-27,31]. The second focus group (n=4) and 3 individual interviews were with PCPs who did not have previous experience with CDS tools for pediatric asthma. The third focus group (n=2) and 1 individual interview were with health care IT professionals who had previous experience developing ambulatory CDS tools. Focus groups and interviews were conducted over Zoom (Zoom Video Communications) or in person in clinic workrooms. Focus groups and interviews were facilitated by a PhD-level, certified asthma educator with expertise in clinic- and school-based asthma management intervention (JH [female]) or a PhD-level clinical psychologist with expertise in mobile and eHealth asthma interventions (DAF [male]). Both study authors have experience in conducting qualitative interviews with families of children with asthma and medical providers. Participants were made aware of the goals of the study during the informed consent process and the interviewers briefly summarized their professional expertise in pediatric asthma. Participants were compensated US $50 for their time. In addition, a brief demographics survey was distributed during each focus group.

Focus groups and interviews were conducted in a semistructured format, with separate guides developed for PCPs and health care IT professionals. Sessions ranged in length from 25 to 65 minutes, with an average length of 38 minutes. The PCP interview guide included open-ended questions on how the provider currently diagnoses and treats asthma, if they use any validated tools for diagnosis or treatment planning, familiarity with NAEPP guidelines, how a CDS tool embedded in the EHR might help them provide guidelines-based care, and what functions could be useful in such a tool. The health care IT professional guide included questions on design and implementation processes of CDS tools into the EHR, best practices for collaboration with research and clinical teams, and strategies for evaluating and maintaining CDS tools across multiple clinics.

Focus groups and interviews were audio-recorded, transcribed, and then analyzed by 3 coders (DAF, JH, and JLM) using NVivo 14 (Lumivero). Field notes were taken during data collection. We used an inductive approach by coding data based on similar topics and content across transcripts, clustered by PCPs and health care IT professionals, to develop themes. Themes were derived from overlapping content between PCPs’ data and included descriptions of assessments conducted to make an asthma diagnosis, previous attempts or opportunities to implement updated NAEPP guidelines, and how a CDS tool could be implemented using the EHR and fit into current asthma management workflow. Themes from overlapping content between health care IT professionals’ data included discussion of processes used to design CDS tools (eg, initial assessment of required functionality, testing mock designs to evaluate alignment with PCP workflow) and strategies to collect evidence that indicated a tool’s value to a practice and the broader health system. We then collapsed themes into 2 domains, Current Practice and CDS Tool Development. The 3 coders then used a final codebook that described the themes in each domain to analyze all transcripts (Table 1) independently. Codes for each transcript were then discussed and modified collectively until consensus was reached.

**Table 1:** Qualitative codebook for interviews with primary care providers and information (for technology health professionals).

Domain and code		Definition
**Current practice**		
	PCPs^a^ describe their asthma diagnosis process and workflow	Description of assessments PCPs conduct to make an asthma diagnosis.
	PCPs describe asthma follow-up care	Descriptions of symptom monitoring at follow-up visits.
	Barriers to asthma management	Mentions of barriers to making asthma diagnosis and to monitoring symptoms at follow-up visits.
	Asthma action and treatment plans	Statements about how asthma treatment regimen is communicated to families.
	Opportunities to implement new guidelines	Statements about barriers to implementing new guidelines and explanations of previous attempts to implement them (individual PCP or practice-wide).
**CDS^b^ tool development**		
	How can a CDS tool fit into provider workflow	Recommendations on components and functionality of ideal tool, including patient surveys, how responses are displayed for the provider, how treatment or prescription options are displayed for the provider.
	How can a CDS tool be best designed	Recommendations on design strategies and examples of tools that were successful or unsuccessful in having the components or functionality that providers wanted and used.
	Strategies to evaluate impact of a CDS tool	Explanations of how to gather evidence needed to demonstrate tool’s value to a practice and the institution or health system. Mentions of pipeline through which projects move forward.

^a^PCP: primary care provider.

^b^CDS: clinical decision support.

### Ethical Considerations

The institutional review board at the University of Florida approved all study procedures and measures (IRB 202202480). All individuals provided informed consent before participating in the current study and were reimbursed US $50 for their time. All study data presented below are deidentified.

## Results

### Participant Demographics

Across the 3 subgroups, 11 participants reported their gender as women and 4 reported as men. Participants reported their race as White (n=11), Black or African American (n=1), or Asian (n=3), and 2 reported their ethnicity as Hispanic or Latino-a-x. To account for providers with multiple racial or ethnic identities, participants could select more than 1 race or ethnicity response such that the total n and percentages summed to greater than 15 and 100%, respectively. PCPs using a CDS tool had an average of 14.6 (SD 13.9) years in practice, while those not using a CDS tool had an average of 16.1 (7.6) years (Tables 2 and 3).

**Table 2:** Demographics of study participants.

Characteristic		Participants, n (%)
**Gender**		
	Men	4 (27)
	Women	11 (73)
**Race or ethnicity^a^**		
	White	11 (73)
	Black or African American	1 (7)
	Asian	3 (20)
	Hispanic or Latino-a-x	2 (13)

^a^Participants could select more than one race or ethnicity response.

**Table 3:** Demographics of study participant subgroup.

Participant subgroup	Years in practice		
	n	Mean (SD)	Range
PCPs^a^ with asthma CDS^b^ tool experience	5	14.6 (13.9)	3-36
PCPs without asthma CDS tool experience	7	16.1 (7.6)	3.5-27
IT professionals	3	—^c^	—

^a^PCP: primary care provider.

^b^CDS: clinical decision support.

^c^Not available.

### Current Practice for Asthma Management

Within the Current Practice domain, five themes were developed, that are (1) description of asthma diagnosis processes, (2) asthma follow-up care, (3) barriers to asthma management, (4) asthma action or treatment plans, (5) opportunities to implement updated NAEPP guidelines (refer to Table 1 for full codebook and definitions of themes). Quotations from participants in each of the three subgroups are identified as (1) PCPs A through E for those with asthma CDS tool experience, (2) PCPs 1 through 10 for those without asthma CDS tool experience, and (3) IT professionals 1 through 3.

#### Diagnosis Processes and Follow-Up Care

PCPs currently implementing a paper-based asthma CDS tool described a patient-reported survey to guide them in making an asthma diagnosis. They described assessing asthma control with the Asthma Control Test (ACT) during visits for specific patient ages (eg, 4 and 8 years) or on designated days of the week. Importantly, these PCPs discussed variable CDS tool implementation and workflows across clinics. Providers expressed the ease of conducting follow-up care using the ACT, which is one of the validated measures included in the CDS tool. The ACT is a validated measure of asthma control and gives providers an objective measure, making overall workflow more standardized and efficient.

It’s much easier every time [to use a CDS tool]. Implementation science tells you that you know just when it’s called for. That’s part of why we do the Asthma Control [Test] at every visit. Instead of somebody having to figure out whether the patient should be diagnosed with asthma or not, and the symptom severity level.

PCP C (with CDS tool experience)

Alternatively, PCPs without experience using an asthma CDS tool described their diagnosis and follow-up processes as based primarily on symptom presentation**,** including recurrent cough, nighttime cough, chest tightness, shortness of breath, and wheezing, with some using structured observation to assess treatment response for acute exacerbations (ie, monitoring symptoms in clinic after administering albuterol) or level of control at follow-up visits. In total, 3 PCPs mentioned using recommendations, such as those published yearly by GINA (Global Initiative for Asthma) to inform their diagnostics.

*The GINA guidelines…Yeah, it’s what I know, and it’s quite updated, and I like it, and I’m comfortable. But I will tell you it’s not the easiest thing to do, especially in the clinic setup. Usually in my previous practice, we had a lot of things that we’re clicking on, and you know it sort of generates for you at least a good estimate. And the reason why I say this because I only have 20 min to do, you know…Even if I suspect that this kid is asthma, after 10 minutes registering and getting roomed in, I only have 10 minutes to figure this out so.* [PCP 7 (without CDS tool experience)]

It was also apparent that PCPs without experience using an asthma CDS tool had knowledge of national and global asthma care recommendations but relied on a more general familiarity with recommendations rather than using a specific tool to aid in their adherence.

I probably should do something, but I usually just ask questions to kinda get an idea of how poorly they are controlled, based on how many times they are using it [quick-relief medication]. Basically going through the guidelines, but not as complete.

PCP 10 (without CDS tool experience)

#### Barriers to Asthma Management

All PCPs endorsed time and workflow management as barriers to managing asthma care and adhering to guidelines consistently and thoroughly.

I would say the main limitation for this and any guideline or anything else we do in clinic is just time. Cause there’s so much to cover in such a short period of time, and trying to prioritize the most important things and spend the limited amount of time that we have on those. Because the most important thing is, albuterol whenever you’re wheezing, and then for the kids that are on steroids, to use them consistently, to be able to emphasize that in a way that it will happen whenever they leave. So that’s a hard thing to do.

PCP 4 (without CDS tool experience)

Among PCPs using a paper-based CDS tool, needing to transfer information from paper documentation into multiple EHR modules (eg, progress note, prescriptions, after-visit summary, and patient instructions) was reported as a barrier.

The scoring, writing your impression, especially the [patient]survey. And then you’re gonna, you know, basically do the same thing again, or some similar variation of it in the note or in the chart for sure it’s like it’s definitely work.

PCP A (with CDS tool experience)

Potentially missing asthma diagnoses due to the lack of a CDS tool at the point of care was also identified as a barrier to management.

Missing the possible, not the ones that are established. For the ones you’re considering a new diagnosis. It’s a little bit out of sight out of mind. So people aren’t thinking about [the CDS tool] to administer the survey, and that’s a little bit cumbersome to go and try to find it.

PCP A (with CDS tool experience)

Although PCPs not using a CDS tool described prioritizing the most important aspects of management in their verbal communication with families, they also reported gaps potentially caused by a lack of standardized and accessible treatment plans. In addition, 3 of these PCPs noted insurance coverage of medication costs as influential on their prescribing decisions.

We typically just tell them the medications, and which one is which. You know it’d be nice if I had some kind of chart with all of them on there. The parents, and they usually do get confused. Is it the orange one? Is it this one? So, but I try to go over it with them, which one is rescue, which one is the daily medication. But yeah, we don’t have examples of them in the clinic.

PCP 10 (without CDS tool experience)

So, the biggest barrier that I’ve had is like people that get insurance denies like Flovent versus QVAR…I’m not even sure what the insurance wants. Then, the weirdest things is they want to replicate for kids, like that’s the only one they’ll approve, but it’s for a kid that can’t even use it. Like the barrier, I would say it would be cost, and then sometimes instead of doing like a low [dose]… sometimes the insurance will only cover like a high dose. And so you’re doing it [a higher dose] instead of like a lower dose twice a day. But you’re just hoping that the overall works out to be okay. You have to base on cost. And so I can think of one [patient] in particular that we’ve had to change it based on cost.

PCP 5 (without CDS tool experience)

#### Asthma Action Plans

All PCPs reported using treatment plans to communicate with families but used variable methods to accomplish this task. The use of asthma action plans that are printed or sent to patients via a patient portal was reported by the PCPs using a paper-based CDS tool. Sending additional copies of the treatment plan was described as a useful function.

The majority of families that I have that are actually following their treatment plan. Aware of it adherent usually have a paper copy, that is probably on the fridge or something like that…I do appreciate that if it [the treatment plan] was not done, or if they need another copy or a nurse needs another copy, I can very quickly send it to them again [via patient portal].

PCP A (with CDS tool experience)

Furthermore, 3 PCPs commented that confirming if a treatment plan documented on paper was up-to-date was difficult and may be easier with an EHR-embedded tool that could track treatment plan documentation over multiple visits*.*

You don’t know when it [the treatment plan] was updated you have to search and dig, to find out when it’s updated…And then there’s the prescribing piece where you’re writing all this out, and then you get to do it again out in the prescription module.

PCP C (with CDS tool experience)

PCPs not using a paper-based CDS tool indicated using different strategies to communicate treatment plans, for example, with inhaler labels, verbal explanations of which medications to take when, visual aids, and treatment plan templates from previous training. Some clinicians also used EHR-based tools, including personally developed or borrowed dot phrases (ie, preformatted content that can be pulled into a clinical note using an abbreviation or word), but the type of tool and use differed between PCPs.

I have a dot phrase for an asthma action plan, which I feel like I do more with ones that have controller medicine.

PCP 2 (without CDS tool experience)

I do mostly verbal. Sometimes we’ll look up pictures of the different inhalers on the computer just to make sure that they know which one is red, which one is orange, and how can they use it. And then I have like a PDF of an asthma action plan, but I don’t use it as often as I probably should.

PCP 4 (without CDS tool experience)

Yeah, it’s a smartphrase from a colleague of mine who she got from her attending when she was a resident… I really like the colored ones, because it’s more, if it looks good, the parents will read it…So, it’s just a lot of typing.

PCP 7 (without CDS tool experience)

#### Opportunities to Implement Guidelines

Several PCPs not using a CDS tool referenced awareness of national guidelines and intention to use in practice but noted relying primarily on how they were trained. More experienced PCPs acknowledged that they may be most familiar with previous versions of guidelines while both newer and more experienced providers expressed a desire for training on more recent guidelines.

You know, it’s probably the guidelines that I learned … when I finished residency. It is what was ingrained in me. So, I probably, I know the guidelines changed a few years ago where you can use controller meds more intermittently. I still struggle to wrap my head around that concept.

PCP 1 (without CDS tool experience)

We do have grand rounds every week. That would be a good avenue that I think of. Or you know, to make it a bit more personal level, like someone can go to the clinic. We do have provider meetings every second Thursday. It will be great if someone could, because you know we’re general. We’re the gatekeepers. With all the guidelines you can barely keep up, so it may be a bit of a spoon-feeding, but I mean it’s part of you know provider support.

PCP 7 (without CDS tool experience)

I know GINA just had a recent update, so I looked through it, but I wouldn’t say that I remember everything that’s on it. I would definitely look over it to see what the updates were. The biggest thing I got out of it, because there’s a lot of stuff that’s beyond what I would do as a primary care physician in peds. But I remember the biggest change I think, was using an inhaled corticosteroid as a rescue inhaler. But I don’t know how many people are actually following that, at this point. To be honest, I haven’t really even started doing that, either. It’s hard to change your ways.

PCP 10 (without CDS tool experience)

### CDS Tool Development

Within the CDS Tool Development domain, 3 themes were developed based on health care IT professionals’ and PCPs’ responses. First, how CDS tools can fit into provider workflow; second, best practices for CDS tool design; and third, strategies to evaluate the impact of CDS tools.

#### CDS Tool Fit With Provider Workflow

Health care IT professionals emphasized the importance of provider perspectives in design processes to promote implementation that aligns with realistic clinic workflow. The discrepancy between providers’ initial conceptualization of a CDS tool and the clinical outcomes they seek to improve was highlighted:

What we find often, I think, is that providers will come to us saying they want something but, when we actually talk with them, have a conversation with them about what they’re trying to accomplish, it turns out that what we can do for them is completely different than what they thought they originally wanted.

IT professional

PCPs using a paper-based CDS tool provided examples of how they might translate their processes into the EHR, noting opportunities to reduce providers’ time and documentation burden. They also suggested sending asthma questionnaires to patients via patient portal before their appointment to inform diagnosis and treatment planning but commented that patient response rates would be a limitation of this strategy.

Going from your script to the form… it’s a lot of effort to sort of click your way through. If doing all that work to make a treatment plan could then just generate my script at the same time.

PCP A (with CDS tool experience)

This paper ACT [Asthma Control Test], I generally actually think it’s distributed pretty consistently. What I find is it’s often done during the visit, so I’m not necessarily looking at it to the very end, or I’m asking them to finish it. So we can keep our conversation. If there were a way that they could, have the ACT done prior. We have some of our pre-visit questionnaire and then surveys that we that we do. They can access ahead of the visit. Not that a lot of them take that. But I think if there were people that were more willing to take that up, it would certainly be very helpful to them.

PCP A (with CDS tool experience)

And then it [the questionnaire] pops into Admin. It says you know they have asthma and you’re pulled right in with your smart phrase, and you can review it right there, the scores and everything.

PCP D (with CDS tool experience)

Importantly, PCPs indicated a willingness to modify their workflow to include a CDS tool if embedded in the EHR. Specifically, PCPs mentioned EHR functions they believed could be useful, including reminders of established diagnoses, treatment options, progress note templates, and handouts for families.

If they are known asthmatics, it would be good if there would be an automatic bullet, and I mean I don’t have to look for it, I don’t have to type it, it’ll remind me that hey this kid is asthmatic. In terms of diagnosing, it will be great to have something that we can just click, click, click, and maybe at some point something that they can fill out as well. Especially if they come for wheezing or cough. Then it [the EHR] can all summarize it for us. So that you know it’s a more realistic, doable approach. Everything is on our shoulders.

PCP 7 (without CDS tool experience)

*I like a good order set that kind of spell things out a little bit. I think especially for these updated, like possible treatment options to be reminded of that, of what the options are, and what these new guidelines are.Cause I think otherwise without that reminder, I’m just going to fall back on what I was trained to do the last three years.* [PCP 2 (without CDS tool experience)]

It would be cool if it was embedded in a note template. Everybody tends to use the same well child check templates, and then we all make little tweaks on it. But if there is a standard asthma template, and I guess if you clicked like the certain things, and then it embedded where they were, and if they needed to step up. It could define. It’s not too much thinking about whether they’re mild, moderate, or severe right. There’s like definitions for this, and so it could quickly like auto populate that sort of stuff and have like something to show the parents.

PCP 5 (without CDS tool experience)

#### Best Design Practices

Health care IT professionals discussed the importance of using tools the way they are already designed within the EHR, which often requires first understanding current workflows and then guiding clinicians from their usual practice toward a modified workflow that accomplishes clinical goals while being compatible with existing functionality. Compromise between individual PCPs’ nuances in how they work and new workflows can be challenging. As such, they indicated that regular communication with a designated physician champion for the implementation of a new CDS tool is paramount to an effective design process and subsequent clinician buy-in.

…we ask them, how are you doing this today?... Sometimes they’ll ask for something that is totally different than the process they’re following today. They may be asking for this because they want to simplify things, but they may be asking it because they’re in that mindset of oh, I’m electronic now, and the way we did it before isn’t applicable at all. But that’s not necessarily the case.

IT professional 2

We are almost always asking them to do something different. So even if it is, for instance, sending out some sort of inventory, filling out a form, they still have to navigate to a place to get to that form and open it. It is that sort of moving them from what they’re doing now to something completely different, even though it potentially has a lot of benefit for them.

IT professional 1

The health care IT professionals also provided an example of mechanisms for different CDS tools, such as pulling data from multiple records into a health maintenance summary to track chronic care (eg, routine screenings), drug dosing calculators within the medication ordering processes, and best practice alerts (BPAs) using in conjunction with questionnaires to guide decision-making. Importantly, they highlighted that even though clinicians may envision a new tool as a BPA pop-up, the risk of alert fatigue may compromise the potential utility of the tool in changing clinician behavior as well as negatively impact other CDS tools.

We’re trying to really integrate our data analytics group generating reports to say, “How are we doing with this clinical decision support tool depending upon the nature of the tool?” We want to see that at least meeting expectations in a changing behavior because if it's not, and we have alert fatigue, then, of course, that also has a deleterious effect on all the other local decision support systems we put out there.

IT professional 3

We don’t just want to encourage people to make BPAs for everything, because ultimately they get ignored, and so there might be some other tool. Understanding exactly what they are wanting to do and where they envision this working in their workflow.

IT professional 1

Finally, the health care IT professionals emphasized consideration of how EHR changes may affect a broader health system outside of the clinic implementing a new tool.

Sometimes an ask will be something that we need to set at what we call the system level. Which means it would impact users at [other clinic sites]…And because of the way they practice, which sometimes can be completely different, we have to come up with either work around where we don’t have to set something at the system level, which those aren’t always available.

IT professional 2

#### Evaluation of Impact

Working closely with a physician champion to share a comprehensive understanding of existing workflows and potential changes informs evaluation of a new CDS tool. One health care IT professional described how workflow events (ie, discrete steps a provider is taking in the EHR during an encounter) must be captured in a reporting system to reflect whether the tool is supporting physician decision-making behavior in an intended manner. Demonstrating impact to key stakeholders, for example, health system financial leadership, physicians being asked to implement a new CDS tool, and other clinical professionals working alongside them, was discussed as essential to willingness to change usual practices. Considering impacts on the broader health system was again highlighted as critical for regulatory reporting, a standard aspect of CDS tool implementation.

I ask the question about how do you want to demonstrate to your stakeholders that you’ve made an impact. So how are we going to be able to report on this and show, what are you going to measure, to demonstrate that it’s efficacious, so that they can then sell this, so they can get people on board to follow. And use the tools we’ve implemented.

IT professional 1

We must know that workflow, because at certain points those key workflow events can be captured in a reporting system that way. We know that whatever report we have is reflective of the workflow to make sure that we’re indeed doing the right thing.

IT professional 3

There’s also differences within the various departments, various workflows, not just among clinicians among departments that we may be wanting to implement a tool that crosses, so that from a from a maintenance standpoint we have a single tool that we can use to collect data for across the organization and report out in one place. We must make sure that tool is going to work for everyone, and they still yet all those results and information still funnels out to the same place.

IT professional 1

## Discussion

### Principal Findings

The goal of the current study was to inform the design of an EHR-embedded CDS tool that adheres to the 4 pillars of NAEPP guidelines by gathering qualitative data from target stakeholders (ie, PCPs and health care IT professionals). Overall, PCPs described a variety of barriers to consistent guideline adherence during their care of children with asthma. Commonly endorsed barriers included having limited time during encounters, which was linked to inconsistency in the assessment of asthma symptoms and inconsistent provision of asthma treatment plans. Furthermore, PCPs described current difficulties with integrating guidelines into their workflow and that searching for documentation tools or printed resources was often challenging. These data are consistent with previous research showing that PCPs regularly encounter extrinsic barriers that limit their ability to adhere to asthma guidelines [20]. Table 4 summarizes the key themes and conclusions that EHR-embedded CDS tool can improve care and guideline adherence.

**Table 4:** Summary of key themes and conclusions from qualitative interviews.

Theme	Conclusion
Limited time leads to inconsistent assessment of asthma symptoms and variable communication about treatment plans depending on provider	EHR^a^-embedded CDS^b^ tool can improve care through efficient, standardized symptom assessment and diagnosis at the point of care and templates and resources to optimize provision and documentation of consistent treatment plans
Providers are aware of national guidelines but lack supports to implement them in practice	EHR-embedded CDS tool can improve guidelines adherence through structured support throughout clinical encounter to promote guideline implementation and fit within existing clinical procedures

^a^EHR: electronic health record.

^b^CDS: clinical decision support.

Data from this study suggest that CDS tools were positively viewed by PCPs and may be beneficial for overcoming noted barriers to improve provider adherence to NAEPP guidelines. PCPs who have previously used a paper-based CDS tool described that it helps them more readily identify new asthma diagnoses, improves their adherence to several NAEPP guidelines (eg, assessment of asthma control and provision of treatment plan), and were enthusiastic about its implementation in the EHR to improve asthma management. Similarly, PCPs who had not used an asthma-focused CDS tool identified several areas where such a tool could be beneficial to improve adherence to NAEPP guidelines (eg, improving consistency in provision of treatment plans) and expressed interest in incorporating a CDS tool into the EHR. These findings align with previous studies demonstrating that CDS tools improve provider adherence to national asthma guidelines [20] and are viewed favorably by PCPs [21,32], suggesting that CDS tools may be an especially viable mechanism to increase PCP adherence to NAEPP guidelines.

A notable change in recent guidelines is the inclusion of SMART [7]. SMART shows promise in reducing asthma exacerbations among those individuals with suboptimal asthma control [33]. However, although PCPs who did not currently use a CDS tool for asthma management indicated a general awareness of new guidelines, they acknowledged often relying on how they were originally trained to treat pediatric asthma, being unfamiliar with key changes to guidelines, and having limited professional development opportunities to familiarize themselves with updated guidelines. Thus, an important takeaway from the current study was that integration of an asthma management CDS tool into the EHR may facilitate adoption of SMART and be especially timely [30].

A unique aspect of this study is that we purposely included health care IT professionals to gather their perspective on how to best integrate a CDS tool into the EHR. Overall, their feedback echoed best practices for the development of CDS tools, which include designing for the end user’s context in mind and providing the right information at the right time in the PCPs’ workflow [34]. Feedback from PCPs on the development of our CDS tool aligned with that of that health care IT professionals and largely focused on ways the tool could improve efficiency and consistency. Specifically, PCPs described a desire to have guidance across the encounter to assist with diagnosis, severity determination, assessment of asthma control, and prescribing and ordering in a manner that is concordant with NAEPP guidelines. Furthermore, there was strong interest in support in the EHR for automating the note writing and treatment plan components of the encounter to reduce time-related barriers, improve documentation of relevant asthma-related information, and increase the likelihood that families would be provided an asthma treatment plan.

### Strengths and Limitations of This Study

Our study has several notable strengths. First, consistent with strengths of this study include following best practices for the formative development of EHR-embedded CDS tools, we purposely engaged the end users of our planned CDS tool to provide their perspectives on its need at the point of care, the fit within their workflow, and its potential features. A unique strength of this study is the inclusion of the perspectives of 2 groups of PCPs, those with a longstanding history of using an effective paper-based CDS tool for pediatric asthma and those who have not used CDS in asthma care, across 2 health care systems in geographically distinct areas. This allowed us to gather diverse perspectives on our key research questions. Finally, we also gathered important data from health care IT professionals to understand the opportunities and challenges that scientific teams interested in developing EHR-based CDS tools should consider.

There are several study limitations that should be considered. First, we acknowledge that this study included a modest number of health care providers, all of which represent clinicians from large academic medical centers and use the EHR in their daily practice. Thus, although it is notable that health care providers were recruited from 2 distinct academic medical centers, the findings from this study may not generalize to health care providers with less experience using an EHR or who practice outside of academic medicine. Second, the responses that we received were consistent across individuals, however, we acknowledge that the current study included a small number of health care IT professionals. It is possible that recruitment of additional health care IT professionals would have yielded different opinions or data. Third, the health care IT professionals were from a single academic medical center that uses an enterprise-wide EHR system. Therefore, it is possible that their opinions may not be generalizable to other health care systems or EHR platforms. Finally, participants were largely recruited via convenience sampling–based, introducing some concern for selection bias. We attempted to limit this concern by directly encouraging open and honest responses and ensuring that the study investigator did not have a professional relationship with the participant whenever feasible.

### Conclusions and Future Directions

In this study, we used user-centered design principles to guide a qualitative study on perceived barriers and facilitators to a primary care–based, EHR-integrated asthma CDS tool. Participants in this mixed-methods approach expressed their interest in adopting an efficient asthma management CDS tool that could help them adhere to NAEPP guidelines and improve clinic workflow. Similarly, health care IT professionals perceived having an asthma CDS tool to be useful, as long as it adhered to EHR design standards. Additional recommendations from IT professionals included limiting BPA-alert fatigue and building automated reports to track use of the CDS tool. Thus, future directions will be to design an asthma CDS tool that (1) prompts PCPs in a low-burden approach that limits alert fatigue, (2) guides PCPs in diagnosing asthma and assigning a severity using native EHR tools, (3) provides PCPs with guidelines- and severity-concordant therapies to create an asthma treatment plan, and (4) streamlines follow-up care using EHR-embedded asthma control assessment tools.

## Abbreviations

ACT: Asthma Control Test
BPA: best practice alert
CDS: clinical decision support
GINA: Global Initiative for Asthma
EHR: electronic health record
NAEPP: National Asthma Education and Prevention Program
PCP: primary care provider
SMART: single maintenance and reliever therapy

## Data Availability

The datasets generated during and/or analyzed during this study are not publicly available due to institutional regulations but may be available from the corresponding author on reasonable request.
